# A leap of faith: building the trust in human biobanks

**DOI:** 10.3389/fgene.2023.1261623

**Published:** 2023-10-19

**Authors:** Yi Zhang, Bohua Liao, Ruipeng Lei

**Affiliations:** ^1^ School of Philosophy, Huazhong University of Science and Technology, Wuhan, China; ^2^ The Institute of State Governance, Huazhong University of Science and Technology, Wuhan, China; ^3^ School of Marxism, Center for Ethics and Governance of Science and Technology, University of Electronic Science and Technology of China, Chengdu, China; ^4^ Center for Bioethics, Huazhong University of Science and Technology, Wuhan, China

**Keywords:** human biobanks, trust, trustworthiness, trust environment, leap of faith, bioethics

## Abstract

**Background:** Human biobanks are an essential resource for contemporary medical research, crucial in treating and preventing human diseases and improving health. Public trust in human biobanks is a vital social prerequisite for their continued operation and related research.

**Methods:** Drawing on the “leap of faith” theory proposed by Georg Simmel and Guido Möllering, this paper first examines the relationship between public trust and human biobanks and the process through which such trust is established. Subsequently, based on the results of this analysis, targeted policy recommendations are put forward to consolidate or enhance public trust in human biobanks.

**Results:** Public trust in human biobanks stems from certain “good reasons,” through which uncertainty and vulnerability are “suspended” by faith, leading to a leap toward the “land of expectations.” In this progress, the critical factors in building and enhancing public trust in human biobanks are the public’s propensity to trust, the inherent trustworthiness of human biobanks, and the security and interactivity of the trust environment.

**Conclusion:** Public trust in human biobanks cannot be determined by any universal formula, as it is influenced by many factors, including intangible elements such as faith that defy empirical understanding. Nonetheless, public trust in human biobanks can be enhanced through measures such as fostering the public’s propensity to trust, enhancing the inherent trustworthiness of human biobanks, establishing structural safeguards for the trust environment through ethical norms, systems, and supervision, and promoting public participation.

## 1 Introduction

Trust is widely recognized as a cornerstone of human society, enabling individuals to reduce systems and procedures’ complexity and facilitate daily life. This is particularly true in contemporary times, characterized by pervasive uncertainty, where the role of trust in human life is self-evident. As such, research on trust has attracted considerable attention within academia since the 1950s. Literature review on trust reveals a vast and diverse body of work, with no consensus on its definition to date. This can be attributed to the broad scope of trust and its multiple meanings in everyday usage, as well as to the different perspectives or worldviews from which it is studied, such as those of psychology, philosophy, sociology, economics, and others. D. Harrison McKnight and Norman L. Chervany have likened the process of studying trust to that of “blind men and an elephant (Harrison and Chervany, 2001, pp.27–54).”

Nonetheless, addressing practical issues of trust in daily life is paramount. The analytical examination of trust within specific contexts holds the potential to contribute to the resolution of various real-world challenges where trust assumes distinct yet pivotal roles. Such roles encompass the reinforcement of social cooperation (Dimock, 2020), the promotion of political democracy (Uslaner, 2003), the facilitation of medical treatments (O’Neill, 2002), and the regulation of developments in science and technology (Toreini et al., 2020). Human biobanks (HBs), as infrastructures designed for the collection and storage of human biological materials and data for research purposes, occupy an indispensable position within contemporary biomedicine and public health. The establishment and operation of HBs hinge substantially on public support and active participation. The building and cultivation of public trust in HBs bear substantial practical significance for advancing HBs and enhancing human wellbeing. Moreover, discussions concerning public trust within the context of HBs contribute to a deeper comprehension of the nature of trust. From a practical perspective, trust constitutes a tangible social relationship that pertains to how individuals navigate their existence within the world and coexist with others (Guo et al., 2012).

Drawing upon a variety of definitions pertaining to HBs (Caenazzo et al., 2015; European Commission, 2012, p.12; Conroy et al., 2023), HBs can be delineated as long-term and standardized infrastructures for the collection, processing, and storage of human biological materials and pertinent information. These materials include DNA, RNA, proteins, cells, tissues, organs, body fluids, excretions, *etc.* Furthermore, HBs involve clinical information, environmental factors, as well as data and information resources related to lifestyle factors. Fundamentally, HBs’ function as integrated frameworks that amalgamate biological materials with pertinent information. They can be harnessed within disease treatment, life sciences research, and the development of biological applications across various domains. In addition to conventional sample and data collection and processing methods, contemporary HBs incorporate advanced technologies such as artificial intelligence and big data analysis (Kinkorová and Topolcan, 2020; Narita et al., 2021). These technologies facilitate predictive, preventive, personalized, and participatory (P4) medical research (Bartold and Ivanovski, 2022), positioning HBs as pivotal entities for the future. HBs can be categorized based on their operating institutions, which include academic HBs (public research institutions, universities, *etc.*) (Simeon-Dubach et al., 2020), governmental HBs (local governments, public hospitals, *etc.*) (Lecaros, 2023, p.298; Schmanski et al., 2021), or commercial HBs (state-owned enterprises, biotechnology and pharmaceutical companies, *etc.*) (Dive et al., 2020; Hofman et al., 2014). Additionally, they can be classified based on their funding sources as public HBs (public research institutes, universities, public hospitals, *etc.*) or private HBs (private investments, private companies, venture capitals, *etc.*) (Dive et al., 2020; Hofman et al., 2014).

Given that there are various types of HBs, it is important to clarify that the subsequent discussion is exclusively focused on public HBs. The rationale for concentrating on public trust within public HBs is as follows: (1) A majority of HBs fall within the category of public HBs (Baláž et al., 2022); (2) Compared to private HBs, public HBs are more focused on scientific research rather than obtaining funds or increasing profits. The funds of public HBs mainly come from public taxes, and public support and trust are crucial to their successful operation (Tozzo and Caenazz0, 2020); (3) Public HBs are the biobanks most closely related to the majority of the populace. As indispensable infrastructures within biomedical research, they are essential for public wellbeing, thereby necessitating prioritization of trust-related issues within these biobanks (Gao et al., 2022); (4) The public often has a strong willingness to participate in and donate to public HBs, rendering discussions on public trust in public HBs particularly pertinent (Critchley et al., 2021); (5) In the current era characterized by the prominence of big data, public HBs offer greater accessibility to biological information encompassing clinical data and lifestyle influences compared to private HBs, making their public trust issues more prominent (Pastorino et al., 2019). Thus, building upon a theoretical analysis, this paper endeavors to provide a helpful approach for building public trust in public HBs to facilitate their better development and governance.

## 2 Theoretical foundations of public trust in HBs: a leap of faith

Although trust lacks a precise definition, specific fundamental characteristics are widely recognized by researchers, including the presence of risk and uncertainty (Deutsch, 1958), and the vulnerability (Boss, 1978; Rousseau et al., 1998) and expectations of the subjects towards the objects (Bradach and Eccles, 1989), among others. This section discusses these characteristics and components of trust, using them to elucidate the theoretical underpinnings of public trust in HBs. Additionally, as proposed by Georg Simmel, the mysterious force of faith (McCole, 2005) is also significant as it can account for seemingly irrational trust behaviors. Collectively, these characteristics form the foundation of trust. According to the theory of leap of faith advanced by Guido Möllering, trust is achieved when the subjects have expectations of the objects and are therefore willing to take risks and expose themselves to vulnerability, thereby suspending uncertainty and leaping onto the land of expectation. Guido Möllering vividly describes this process as “trust can be imagined as the mental process of leaping—enabled by suspension—across the gorge of the unknowable from the land of interpretation into the land of expectation (Möllering, 2001).”

The leap of faith can apply to individuals and other types of entities and even abstract principles and systems (Braun et al., 2021). It can also be employed in studying and interpreting public trust in HBs. HBs are defined as “resources constructed for genetic research purposes, including (a) human biological materials and information generated through their analysis; (b) extensive related information (OECD, 2010).” HBs represent a crucial resource for investigating the causes and mechanisms of genetic diseases and devising personalized medical treatment plans. Current research on HBs has already demonstrated their immense value and potential (Kinkorová, 2015). Public trust in HBs entails that the public believes that the operation of the biobanks and the use of samples/data will benefit society and themselves without causing harm, leading them to support the construction of HBs or donate their own biological samples and related biological information. Trust is a powerful and important force in this context, with significant implications for current medical development and promoting human health and wellbeing. Therefore, analyzing public trust in HBs from the perspective of the leap of faith is crucial for maintaining or enhancing public trust in these institutions.

### 2.1 Risk and uncertainty

Risk or investment is widely acknowledged as a necessary condition for trust (Deutsch, 1958; Bigley and Pearce, 1998; Ward, 2006). Trust is future-oriented, with its content consisting of actions that have not yet occurred but which the subjects believe will (the actions that are expected to act by the objects). The future is inherently uncertain, and whether or not the action will ultimately occur is unknown, rendering trust itself a risky endeavor. As Von Niklas Luhmann stated, “The problem of trust therefore consists in the fact that the future contains far more possibilities than could ever be realized in the present and hence be transferred into the past (Luhmann, 1979, p.13),” Uncertainty is a prerequisite for the emergence of trust. Without uncertainty, if the subject possessed complete knowledge and could accurately predict outcomes, this would constitute “control” rather than “trust.” Trust always involves unknown elements, but it cannot be entirely unknown. As Kim Giffin stated, “Absolute zero confidence or totally blind faith in a totally unknown source does not seem to be trust (Giffin, 1967).” Uncertainty represents a state of unknowing, with potential outcomes that may be either favorable or unfavorable. The possibility of unfavorable outcomes, i.e., risk, truly concerns the subjects.

For donors to HBs, donating biological samples such as organs and tissues and granting consent for research to proceed places them in a state of uncertainty. Donors cannot predict whether activities such as collection and research will be ethically conducted as informed. The uses of biological materials and future benefits to themselves and society are all uncertain and unknowable. If improper or unethical actions are taken by the custodians or researchers of biological samples during acquisition, preservation, or research, donors may suffer harm as a result. The same holds true for members of the public who support HBs. They trust HBs but cannot predict whether their research will yield beneficial results or whether they will deviate from their promised direction and cause harm to humans or society by violating ethical norms. Consequently, due to the presence of uncertainty and risk, the subjects must also necessarily be vulnerable. Therefore, due to the existence of this uncertainty and risk, the trust subject must be vulnerable. Of course, this uncertainty and risk must be maintained within reasonable limits, as exceeding the public’s tolerance threshold would undoubtedly undermine the development of trust, leading to distrust instead. Therefore, managing uncertainty and risk within a certain reasonable range is a prerequisite for public trust. From the public’s perspective, a degree of awareness and control over the risks and uncertainties associated with HBs can be achieved through informed consent and the right to withdraw and opt-out. Through informed consent, the public can gain insight into the usage of their samples/data, which not only ensures their autonomy and bodily integrity are not infringed upon but also helps prevent the improper acquisition of their samples/data (Mikkelsen et al., 2019; Sheehan, 2011). When public has the right to withdraw and opt-out, they are empowered to promptly prevent harm arising from unlawful or unreasonable acquisition and research practices (Bhaimia, 2018).

### 2.2 Vulnerability

Trust entails subjects ceding control to the objects and becoming dependent on them. However, the objects’ actions, character, and intentions cannot be confirmed (Seligman, 1997, p.21), an engendering vulnerability in the subjects due to ignorance and uncertainty. Vulnerability refers to the severity of potential adverse consequences or losses, with making oneself vulnerable constituting risk-taking (Das and Teng, 2004; Mayer et al., 1995). In order to achieve trust, these risks must be taken, meaning that vulnerability must be accepted. Whether or not the subjects will take these risks depends on their willingness to bear the risk and accept vulnerability. The public’s trust in HBs and their willingness to donate implies that they have accepted this potential vulnerability. So where does this willingness come from? Generally speaking, low levels of risk perception lead to risk-taking, with this perception being related to the subject’s level of psychological optimism (Mayer et al., 1995). As Guido Möllering explains, “intention to accept vulnerability” does not imply that the subject is willing to be harmed; on the contrary, the subject harbors a highly optimistic expectation that vulnerability is not problematic and will not result in harm (Möllering, 2006, p.9). In other words, the subjects are confident that objects will not betray them and feel secure in relying on them, i.e., they feel safe, reassured, and comfortable (not anxious or fearful) (Harrison and Chervany, 2001, pp.27–54). It can be said that although members of the public may become vulnerable due to uncertainty and risk when donating biological samples or trusting HBs (such as being harmed during sampling or having their privacy breached), their high level of optimism toward HBs reduces their perception of vulnerability due to their sense of security.

### 2.3 Expectation

Expectation can be viewed as the destination of the trust (Evans and Kruger, 2009; Evans and Kruger, 2014; Möllering, 2001), as it is the expectation that motivates a series of potentially risky actions that may lead to the realization of this goal. Expectation is not merely the anticipation of achieving a result, but also an expectation that the objects are trustworthy (Samuel et al., 2022). Trustworthiness, being the well-grounded basis for trust, ensures that trust has a sufficient foundation only when the object is deemed trustworthy. Trustworthiness becomes evident through the object’s capacity and willingness to undertake actions that meet the subject’s expectations (Wright, 2010). As an object of public trust, the trustworthiness of HBs becomes apparent in its capability and willingness to fulfill the public’s expectations, particularly in the ethical and responsible utilization of biological materials and data to attain societal benefits. Nevertheless, it is imperative to acknowledge that HBs, as abstract systems, cannot independently actualize the public’s expectations. Instead, they rely upon the actions of their operational agents, namely, managers and researchers, to translate these expectations into reality. Consequently, a portion of the public’s expectations is transferred onto the competencies and intentions of these operational agents, their trustworthiness manifested in their professional knowledge and skills, benevolence, and integrity (Mayer et al., 1995; Meyer et al., 2008).

The starting point on the journey towards “expectation” is the “land of interpretation (Möllering, 2001),” where individuals may derive “good reasons” based on their everyday experiences. While these “good reasons” appear to provide a foundation for trust, it does not necessarily lead to the destination and cannot determine trust. This is because, on the one hand, the actions of the objects are often unpredictable, and many of objects are unfamiliar or even unknown entities to subjects. On the other hand, these “good reasons” may be based on the subjects’ assessment of the “likelihood” that the objects will fulfil their expectations (Nickel, 2009). This assessment or expectation is more of a value judgment than a factual judgment. For instance, in the context of public trust in HBs, public members are willing to donate their biological materials and data not solely for their own benefit but also to benefit others.

### 2.4 Faith

Georg Simmel observed that the link between the identifiable foundations of trust and the actual expectations of individuals when they attain a state of trust is tenuous (Möllering, 2001). The emergence of trust may not be entirely predicated on rational evaluation, with other elements potentially playing a role, which Georg Simmel referred to as “faith.” This “faith” is enigmatic, representing “a state of mind which has nothing to do with knowledge, which is both less and more than knowledge … It expresses the feeling that there exists between our idea of a being and the being itself a definite connection and unity, a certain consistency in our conception of it, an assurance and lack of resistance in the surrender of the Ego to this conception, which may rest upon particular reasons, but is not explained by them (Simmel and Frisby, 2004, p.190).”

This “faith” embodies high moral value, as if fulfilling expectations is the duty of the objects, and betrayal necessitates utter baseness. Thus, the subjects possess a sense of “certainty” towards the objects, such as a donor’s certainty that HBs utilize their samples/data will not result in harm to themselves. Faith connects “interpretation” with “expectation” and possesses an inherent function of “suspension (Möllering, 2001).” “Suspension” temporarily suspends the uncertainty and fragility, as if it has been resolved. In HBs, public trust in them is largely predicated on an enigmatic emotional response towards science and technology, as well as the researchers responsible for operating and managing HBs. Through this emotional response, a sense of “certainty” appears to be attained, thereby bridging the gorge of “uncertainty.” Once the leap to a favorable (or unfavorable) expectation state has been completed, the trust process continues, with the “land of expectation” becoming the “land of interpretation” and another gorge requiring leaping once again (Möllering, 2001). However, trust remains fragile and possesses reflexivity. Once the subject realizes that their goodwill has been violated, the process is interrupted. For instance, donors’ certainty or faith that HBs will not harm them remains fragile and reflexive (Möllering, 2005, pp.17–36) (once donors discover that their goodwill has been violated or betrayed, the trust will be interrupted).

Based on an analysis of the fundamental characteristics and components of trust, and in conjunction with the “leap of faith” theory, it is possible to elucidate certain mechanisms underlying the operation of public trust in HBs. However, the question remains whether a universal trust formula can be established to achieve public trust in HBs. This article will provide a detailed examination of this issue.

## 3 Is there a universal formula for the construction of public trust in HBs?

Is there a universal formula for constructing public trust in HBs? The answer is no. Firstly, as previously mentioned, the connotation of trust is complex, encompassing potentially disparate types of objects that cannot be homogenized. Secondly, the nature of trust contains an elusive, transcendent element — “faith.” Thirdly, trust is a dynamic process, produced or cancelled through continuous interaction with the subjects, and objects cannot be fixed. Trust does not constitute a fixed response obtained through a certain procedure under a given stimulus. So, is trust entirely elusive? Although we cannot be completely sure, some empirical elements can still be grasped. While these elements do not play a decisive role in trust, positive construction can still exert a positive impact.

As previously demonstrated, the starting point of trust is the “good reason” derived from real-life experience, which leaps to “expectation” through some form of “faith.” Here, “good reason” is empirical and thus may be discernible through certain means. “Faith,” although mysterious and unknowable, can be intuitively judged to be more or less related to the subjects’ propensity to trust, particularly their general attitude towards the objects of trust. “Land of expectation” is the destination but also the starting point for subsequent acts of trust. Once the previous act of trust has been completed, the “land of expectation” becomes the another “land of interpretation.” Therefore, by capturing some characteristics and components of trust based on experience, we may be able to provide a general direction for constructing trust. Public trust in HBs primarily encompasses three fundamental elements: The subjects (public), the objects (HBs), and the trust environment. Each element is directly related to whether or not trust ultimately occurs, mainly manifested as the propensity to trust the public, the trustworthiness of the HBs, and the security and interactivity of the trust environment.

### 3.1 Subjects’ propensity to trust

For HBs, sources of biological samples and data include samples collected during patient disease treatment, clinical medical information, biological information donated by healthy volunteers, and epidemiological information collected through prospective molecular epidemiology and large-scale cohort studies, among others (Winickoff, 2015; Tan and Timpson, 2022). Consequently, the subjects include clinical patients with pathological tissues removed, volunteers who donate biological samples and clinical data, and the general public. The subjects must decide whether to donate their biological materials and information and whether to support research using samples or data, which is critical for the operation of HBs and related research. Whether or not trust is generated is closely related to the propensity of the subjects, which plays a certain role in “faith.” The propensity to trust constitutes a personality trait that determines one’s general view of the natural world. When a trusted object is a person, it manifests as a general expectation of the trustworthiness of others (Mayer et al., 1995); when the trusted object is an abstract entity, it manifests as confidence in its regular operation. When the subjects possess a high optimistic propensity to trust in a certain scientific technology, once HBs and their related cutting-edge research and applications emerge, even without specific experience, the subjects tend to support them. This optimistic general view and expectation may not attain the degree of “faith.” However, it aligns with its direction, representing certainty in something that has not been previously proven, even without basis. Therefore, a high level of optimistic propensity to trust may facilitate the formation of this “faith.” The propensity to trust represents an emotion that transcends cognition but is built on a cognitive foundation. It constitutes a sense of certainty derived from past experiences of the general trustworthiness of technology and related technologies. This sense of certainty is also related to factors such as the sense of security engendered by the effectiveness of institutionalized rules and role expectations for researchers using biological samples.

### 3.2 Objects’ trustworthiness

Although the trustworthiness of the objects cannot be completely sure, their reliability can be understood through direct or indirect experience. We can ascertain some experiential knowledge and characteristics of the objects, which serve as the basis for interpreting trust, i.e., “good reasons.” In this case, the objects are HBs, primarily regarding trust in its operation and related research use. The trustworthiness of HBs can be gauged through their attributes and the reliability of their managers and researchers.

#### 3.2.1 The attributes of HBs

Any emerging phenomenon has its own instrumental value when produced and used, but also entails risks. If it brings a high possibility and degree of harm, and its instrumental value or prospects are not outstanding, then the public will certainly not tend to support it, and it must also be unethical. Therefore, the risk-benefit ratio measurement of emerging phenomena and the actual risk assessment are important criteria for its trustworthiness.

In today’s uncertain society, HBs have immeasurable potential. Contemporary HBs have evolved from small-scale or individual databases into complex and dynamic entities integrated within extensive infrastructure networks (Caenazzo and Tozzo, 2020). Prominent examples include the Biobanking and Biomolecular Resources Research Infrastructure–European Research Infrastructure Consortium (BBMRI-ERIC). It enables people with different professional backgrounds and specialties to collaborate to obtain and collect biological and clinical data from human subjects, provide research-based treatment plans for complex and rare diseases, and provide a foundation for precision medicine and personalized health services (Coppola et al., 2019). Despite the rapid development of HBs, their status as emerging phenomena exposes them to certain limitations, primarily constrained by the current state of technological development and the potential for human misuse. These limitations engender a spectrum of concerns, including human dignity and moral status, safety and security, privacy violations, societal discrimination, and other potential harms. These concerns encompass various facets of HBs, spanning the realms of sample and data collection, processing, and research activities.

For instance, a prominent ethical concern pertains to the commercialization of HBs. The commodification of human biological materials raises intricate questions concerning bodily integrity and self-conceptualization, among others (Boers et al., 2019). Moreover, HBs are currently evolving towards the aggregation of massive datasets. By encoding and digitizing information and data, sharing information and data among HBs has become significantly more convenient, facilitating enhanced cooperation between domestic and international HBs. However, this enhanced accessibility to biological information and data also elevates the risk of information and data leakage, potentially infringing upon the privacy of donors (Dankar et al., 2018). Furthermore, due to biological information’s unique and sensitive nature, certain data elements pertain not only to personal biological information but also embrace familial information. Consequently, the privacy risks associated with an individual’s biological information can cascade and amplify, giving rise to interdependent privacy concerns that extend to entire families or even ethnic groups (Ayday and Hubaux, 2015).

Additionally, the introduction of artificial intelligence and big data analysis into HBs has altered the sample and data collection process. HBs now possess the capability not only to actively collect clinical information but also to extensively gather and analyze the flows of data originating from healthcare systems, social networks, web-based Systems, and socio-economic datasets, among others (Tozzo et al., 2023). As an increasing amount of health-related information becomes digitized, participants and even the entirety of humanity face greater risks, thereby accentuating the salience of ethical concerns related to data security, data misuse, informed consent (including consent for secondary use), *etc.*

The governance and prevention of risks associated with HBs are pivotal in substantiating their trustworthiness. Therefore, a rational benchmark for evaluating the trustworthiness of HBs revolves around the effective safeguarding of participants while concurrently striving to achieve a favorable risk-benefit ratio. An advantageous risk-benefit ratio requires that, generally, the risk to subjects should be minimal, meaning that the probability and degree of anticipated harm or discomfort should not exceed the risks encountered in daily life or during routine physical or psychological examinations or tests (Lei and Zhang, 2022). In situations where research holds the potential for significant societal benefits, it may be ethically defensible and acceptable for subjects to incur risks surpassing the minimum threshold. However, under no circumstances can severe risks or irreversible harm be ethically justified or accepted (Wendler and Miller, 2007). It is worth noting that risk assessment often involves multidimensional considerations encompassing individuals, groups, and society; physical and mental conditions; humans and the ecological environment; domestic, foreign, and global contexts; present and future generations. When evaluating the risk-benefit ratio of HBs, it becomes imperative to consider specific contextual circumstances and use diverse methodologies and perspectives to make assessments and judgments.

With the large-scale datafication of medicine and health, HBs have experienced rapid development. However, the discussion about the risk-benefit ratio related to this has lagged behind (Rychnovská, 2021). There is also limited discussion about the risk assessment associated with collecting large-scale medical and health data, and the few existing discussions focus on the standardized assessment and management of general risks (De Palma et al., 2022). Moreover, despite the objective character of risks, they assume a distinctly subjective dimension when perceived by individuals. This subjectivity emanates from inherent variations in risk perception among diverse individuals, leading to disparities in both the extent and character of risk apprehension, even when confronted with identical risks. Furthermore, an individual’s perception of risk is often influenced by their past experiences and values (Quinn et al., 2013). Therefore, specifically for HBs, the public’s appraisal of the risk-benefit ratio heavily relies on experiential feedback from prior interactions with HBs. In addition, according to the process of the leap of faith, the endpoint of trust (land of expectation) is also the starting point for subsequent trust (land of interpretation). Whether past use of HBs has met public expectations is the basis for continued trust or distrust. If it can become a new starting point for trust, then the gorge of trust can be crossed again.

In addition to its instrumental value, HBs also contain a unique intrinsic value—solidarity. Solidarity here is manifested as establishing HBs for human wellbeing; participants voluntarily undertake social, economic, emotional, and other costs to help others (Ji and Lei, 2019). This is a value concept with the common good, mutual benefit, and altruistic components that are worth pursuing morally. HBs have transformed from a traditional small-scale form in the pre-information age to a modern large-scale form in the current big data age. This value will become more prominent with cross-national and cross-biobank exchanges and integration. This moral value may give the public an innate goodwill towards HBs that transcends cognition and is more emotional. Just as we are more inclined to trust someone with a “benevolent” personality trait, in this sense, it may strengthen the propensity to trust due to solidarity’s value characteristics.

#### 3.2.2 The reliability of HBs’ managers and researchers

The reliability of HBs’ managers and researchers who use samples/data plays a significant role in building public trust. They can be regarded as a group of experts with control and interpretive power over HBs, possessing knowledge and professional skills that are asymmetric with the public. People learn about HBs through them and extend their reliability to HBs.

The trustworthiness of HBs is partly reflected in their managers and researchers. HBs are a non-face-to-face abstract system in the trust process, and the public, as non-professionals, knows almost nothing about it. The continuous generation of its trustworthiness requires face-to-face commitments from the expert group of managers and researchers — “Facework commitments tend to be heavily dependent upon what might be called the demeanor of system representatives or operators (Giddens, 1991, p.41, p.41)” — that is, their reliability. The public’s judgment of their reliability is through traits such as ability, benevolence, honesty, and responsibility (Mayer et al., 1995). Ability refers to whether they have the professional knowledge, ability, and skills to collect, store, dispose of, release, obtain authorization, or conduct experiments and research on biological samples/data in accordance with ethical, scientific, and standards. Benevolence is an altruistic, positive orientation, willing to consider the interests of donors and society as a whole rather than infringing on participants’ interests for personal gain or research purposes or even harming public interests. Honesty is a truthful attitude, whether words and deeds are consistent, whether promises are fulfilled. Responsibility is a willingness to be responsible for the interests of donors and society, consciously following ethical norms and scientific standards in operation. As operators of HBs, they can decide the direction of its use, for good or for evil. Whether HBs can achieve public expectations also depends on the operation of these experts. Experts are a group rather than individuals, and trust in this group is based on society’s general evaluation of this group. Of course, some individuals’ bad actions can also affect the general view of this group, such as the infamous gene-edited baby scandal where Jiankui He severely damaged the image of researchers with his own power (Cyranoski, 2019).

On the other hand, the public’s knowledge of HBs’ trustworthiness comes from experts’ explanations, especially those of these professionals. In public communication, whether professionals will fully inform the real situation, such as actual risks and benefits, and whether they will stand in an objective position and inform risks and benefits with a sincere attitude rather than deliberately emphasizing benefits to facilitate collection and research while reducing risk descriptions or not informing alternative plans. These largely determine the public’s knowledge of HBs, especially their perception of risks and benefits, affecting their judgment of trustworthiness.

### 3.3 The security and interactivity of the trust environment

The security of the trust environment primarily derives from the structural safeguards of the system, such as the rules and regulations and supervision of HBs, legal recourse (Shapiro, 1987), *etc.* These structural safeguards form external constraints by limiting the scope of action and punishing violations, increasing the possibility of the objects following ethical norms, laws, and regulations, improving predictability, and thus reducing the probability of risk to a certain extent, thereby increasing the trustworthiness of the objects. Stable structural safeguards and their effectiveness will bring a sense of security to the subjects, further affecting the public’s general view of technologies such as HBs, acting on the propensity to trust and faith.

Of course, even if the two necessary conditions of propensity to trust of the subjects and trustworthiness of the objects are met, it still cannot necessarily result in trust because trust is essentially dynamic (Mayer et al., 1995), involving a social process in which the subjects and objects interact with each other and with their social context (Nikolova et al., 2015). It is a relational interactive mode. After the trust is given, a still fragile dynamic interaction will occur (Braun et al., 2021). According to the leap of faith theory, uncertainty is suspended through “faith,” trust leaps from the land of interpretation to the land of expectation, and the land of expectation becomes a new land of interpretation for another leap. Due to the mysteriousness of “faith” and the constant change of the “land of interpretation,” there may be continuous and strengthened trust or rapid withdrawal after placing initial trust. It is also possible that after the subjects choose not to trust, the objects or systems reflect on and improve the circumstances that cause distrust, resulting in a result from distrust to trust. Therefore, “suspension” is not a burden that one must bear alone but a continuous construction of mutual expectations and rules (Sydow, 2006, pp.377–392). From this, it can be seen that the interaction between both sides and distrust is essential in the trust process. In the process of social negotiation, distrust marks the possibility of giving voice to those claims that are not heard and not recognized in the social structure (Braun et al., 2021).

Public trust in HBs cannot be established through specific procedures or formulas. It is a mysterious, open-ended result of multistakeholder interaction. However, there are elements through which we can grasp trust. Although it cannot completely lead to a certain result, it can at least develop in the direction of trust to a large extent and further enhance public trust in HBs.

## 4 Schemes for the establishment and reinforcement of public trust in HBs

**FIGURE 1:**
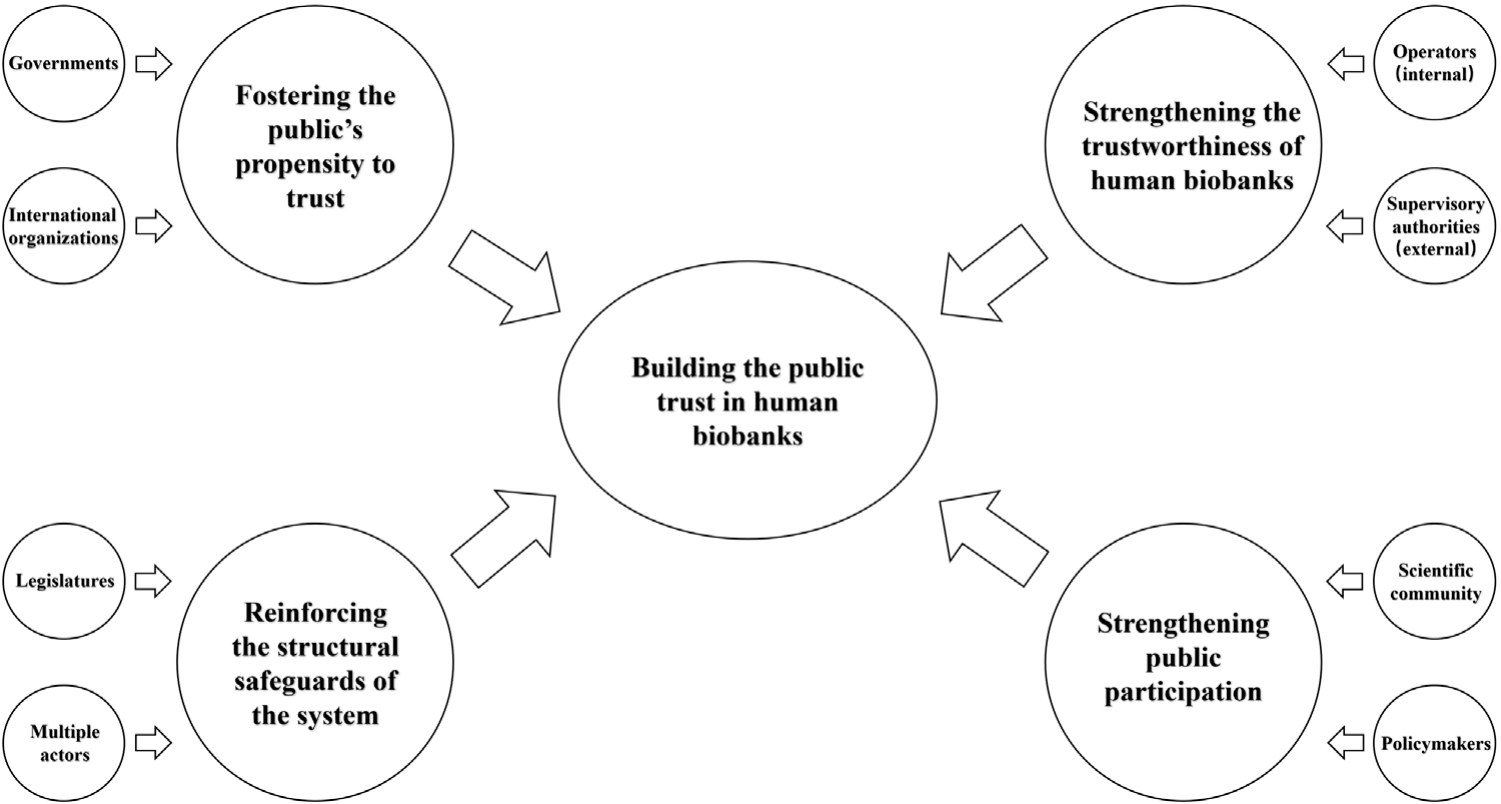
Strategies for building the public trust in HBs.

In contemporary society, the demand for health is becoming increasingly pressing. The role of healthcare is no longer confined to reactive approaches to disease treatment but is progressively shifting towards personalized therapies that emphasize prediction and prevention (Bartold and Ivanovski, 2022). In this context, the importance of HBs as a vital research source is becoming more evident. The swift advancement of sciences and technologies such as bioinformatics, computer science, and big data, coupled with interdisciplinary integration, has facilitated the collection of biological samples and data and enabled more cross-biobank analyses and research (Kinkorová and Topolcan, 2020). However, public support for the operation of HBs, willingness to donate samples or data, and agreement with or support for research using their samples or data hinge on addressing issues of public trust in HBs. Only by establishing and reinforcing public trust in HBs can they develop more effectively and truly benefit society. The path towards building and enhancing public trust in HBs can be pursued by focusing on the propensity to trust of the public, the trustworthiness of HBs, and the security and interactivity of the trust environment. Specific strategies and implementing entities are illustrated in Figure 1.

### 4.1 Fostering the public’s propensity to trust

Fostering the public’s propensity to trust is not solely to support the development of HBs. Indeed, not all applications of science and technology are inherently benevolent. Certain technologies entail considerable risks with minimal benefits, and the malevolent aspect of technological dual-use may be exploited to inflict harm upon humanity and society. Unquestioning trust in such technologies may result in catastrophe. Consequently, it is imperative to cultivate positive factors and an atmosphere conducive to the public’s propensity to trust, encompassing the establishment and enhancement of technical norms and institutional safeguards, responsible scientific research, and the ensuing sense of security within social systems. This entails the following recommended measures: (1) Establishing and refining a system of scientific and technological norms with rigorous standards; (2) Improving scientific and technological legislation and regulatory frameworks, intensifying accountability and penalties for transgressions and abuse; (3) Enhancing systems for apportioning benefits and burdens; (4) Nurturing a culture of scientific integrity throughout society; (5) Fostering a spirit of solidarity among the public; (6) Creating an institutional environment sensitive to trust issues and fostering public awareness of such issues (Tozzo et al., 2023).

These proposals are directed toward governments (specifically, the science and health sectors) and international organizations (such as the World Health Organization, UNESCO, and the United Nations). It is hoped that through these measures, public confidence in HBs can be elevated, enhancing their willingness for altruism and pro-social tendencies (Grežo and Sedlar, 2023), thereby stimulating trust-building actions.

### 4.2 Strengthening the trustworthiness of HBs

The trustworthiness of the objects serves as a crucial foundation for “interprets” and determines whether there is a “good reason” to trust. The bolstering of public trust in HBs can be achieved through both the HBs themselves and their operators (managers and researchers).

Concerning the HBs themselves, the establishment of their trustworthiness primarily centers on risk management. Risk management concerns primarily manifest in the safety and privacy issues of samples/data during collection, storage, access, and release, as well as the potential harm resulting from the collection and utilization of sample data (Takai-Igarashi et al., 2017). In addressing these issues, risks should be minimized to the greatest extent possible while also considering their necessity through comparative analysis. For instance, data security concerns can be mitigated by enhancing the development and implementation of confidentiality technologies to prevent leakage, theft, and unauthorized access; privacy concerns necessitate obtaining informed consent and devising suitable consent procedures, employing anonymous methods to handle samples and data, and appropriately managing genetic information pertaining to entire families; harm to participants and society during collection and use can be diminished through ethical norms and regulation; regular and compliant operation of HBs demands fortifying team management and internal management system construction. Thus, can risk minimization justify the employment of biological samples and data? Risks are an inevitable component in addressing certain diseases and health issues. What is crucial is the risk-benefit ratio. In determining the feasibility of a solution, its net risk must be taken into account. Consequently, a solution-focused risk assessment methodology is required to compare the risks and benefits of various alternative solutions to ascertain whether utilizing biological samples and data for research constitutes the optimal solution (Finkel, 2020); even if it is not the optimal solution, it may still be worth developing under a regulation rather than suspending or even prohibiting it. The responsibility for regulating the risks associated with HBs primarily falls upon the operator’s self-adherence to operational standards, ethical norms, and internal regulatory mechanisms. Simultaneously, external oversight and supervision are exercised by supervisory authorities.

The operators’ reliability constitutes the most immediate source of public perception regarding the trustworthiness of HBs. The responsibility for shaping the operators’ reliability stems from internal factors within the operators’ community and external regulatory and oversight bodies. The reliability of HBs operators can be fostered through several means: (1) Providing professional knowledge and skill courses/training in sample/data collection, storage, release, *etc.*, to augment their professional capabilities; (2) Regularly conducting ethics training and lectures to reinforce ethical principles of integrity and responsibility; (3) Instituting an accountability system to hold accountable those who engage in deception or irresponsible behavior, compelling practitioners to operate with honesty and responsibility; (4) Administering professional qualification certification to elevate the threshold for professionalism and professional ethics awareness; (5) Establishing and refining a curriculum system for bioethics (encompassing clinical ethics, research ethics, and public health ethics) at the higher education level (Lei et al., 2019). In addition to this, researchers should also be conversant with and adhere to pertinent norms for medical research involving humans and clinical trials. However, as medical research progresses and understanding deepens, such norms are continually adjusted and updated. Hence, relevant education should also be conducted on the latest norms and guidelines for relevant research.

### 4.3 Reinforcing the structural safeguards of the system

As previously noted, structural safeguards can enhance the trustworthiness of HBs and provide a sense of security for the public, thereby augmenting the likelihood of trust. Structural safeguards pertaining to HBs can be fortified through ethical norms for management and research, legal provisions, and supervision.

Ethical norms delineate “what (not) to do,” “what should be prioritized,” and “how to do it,” rendering the improvement of ethical norms for HBs essential for guiding management and research actions. The development of ethical norms mainly relies on multiple actors, such as common agreements within professional associations or on declarations and guidelines issued by non-governmental organizations. The formulation of ethical norms must adhere to the following principles: (1) Beneficence/non-maleficence. When formulating ethical norms and standards, the interests of participants must be accorded primary consideration. When promoting social benefits such as medical progress conflicts with safeguarding the value standards of participants, protecting participants must always take precedence. Beneficence/non-maleficence entails minimizing risks and maximizing benefits. (2) Respect. Respect for participants’ rights to life and health, autonomy, and their right to determine the donation and utilization of their biological samples and data. Collection, disposal, and utilization necessitate their informed consent. (3) Justice. Justice is manifested in the equitable distribution of benefits and burdens, encompassing fair inclusion/exclusion of participants and fair access to research results. Further criteria for each research stage based on these three principles should be formulated in light of specific circumstances, with specific regulations devised according to different types of research.

Laws and regulations serve as a fundamental norm, specifying “what is permitted” and “what is prohibited” and being able to curb violations in HB-related activities effectively. Currently, there is a need for ongoing improvement of legislation concerning HBs. Many developing countries either lack specific legislation for HBs or have yet to establish dedicated legislation. In the meantime, the development trends of HBs are increasingly globalized, networked, and intelligent, with cross-border and transnational circulation of samples/data becoming prevalent. Therefore, it is essential to enhance global legislation related to HBs, prompting legislative bodies in developing countries to establish specialized legislation for HBs. Simultaneously, the construction and implementation of transnational and globally compatible legal norms should be pursued.

Supervision constitutes a crucial means of ensuring that actions are ethical, legal, and compliant, playing a valuable role in ensuring the reliability of HB-related activities. Supervision is not merely an internal matter for the scientific community or solely the responsibility of government agencies. However, it necessitates the participation of multiple shareholders, including ethical review by ethics committees, administrative supervision by government agencies, peer review within the scientific community, and public scrutiny through public participation. The ethical review serves as an important means of supervising professionals to operate in accordance with ethical norms (Liu et al., 2020); as enforcers of laws and regulations, government agencies wield considerable deterrent power over HB-related activities through their coercive administrative authority to penalize actions that contravene regulations; peer review constitutes an important means of self-supervision within the scientific community, regulating related activities through self-management and self-correction; public scrutiny involving public participation, media, non-experts in the field, and social organizations can supplement relevant provisions of norms and laws from a broader perspective beyond professional limitations while also exerting public opinion pressure on actions that violate ethics and laws due to its sizable constituency.

### 4.4 Strengthening public participation

Trust arises through interaction. Thus, to bolster public trust, the public must interact through efficacious communication and consultation. In this context, the public encompasses various stakeholders, including patients, participants, and ordinary citizens (Caenazzo and Tozzo, 2020), while the responsible parties for interaction include biobanks, the scientific community, and policymakers.

The interaction process allows the public to continually gain more understanding and knowledge about HBs. The accumulation of understanding and knowledge enables the public to have a more comprehensive perception of the trustworthiness of biobanks, constantly acting on the “land of interpretation.” On the other hand, scientists and other professionals tend to perceive the world in a specific manner, employing particular terms and thoughts within their professional background, potentially narrowing their considerations and choices (Wirz et al., 2023). The public possesses unique local knowledge, and public participation in discussion and consultation may engender a synergy that prompts professionals to make accurate judgments and choices, thereby enhancing the trustworthiness of HBs. Therefore, biobanks and the scientific community can establish a Community Advisory Board to provide consultation and ensure the sharing of information with the public at various stages of the operation of HBs (Luna Puerta et al., 2020), providing effective ways for the public to understand and participate in HBs.

In addition, the public should not be viewed solely as providers of samples/data. Governance and oversight of HBs cannot proceed without public participation (Lensink et al., 2022). Policymakers and relevant authorities must place special emphasis on providing opportunities and channels for communication and consultation with the public regarding the collection of biological samples and data, research, and the application and distribution of research results. Institutional mechanisms should be put in place to facilitate this participation. The interaction between the public and responsible parties, such as government agencies, is a process where stakeholders express their intentions and value judgments. During this process, consensus is formed through communication, which is of significant importance for the public’s willingness to trust. In order to achieve this, authorities can create convenience for public participation through methods such as soliciting public opinions, organizing citizen workshops (Gille et al., 2020), and conducting public hearings, *etc.*

Finally, all responsible parties should adopt a dialectical perspective and value the mistrust that arises from public participation. These questions and doubts can be instrumental in prompting introspection among responsible parties. Reintegrating the demands and rights of the public into consideration can lead to corrective actions and institutional improvements.

## 5 Conclusion

In summary, public trust in HBs is not determined by a universal formula. Numerous factors influence it, including enigmatic elements such as faith that elude empirical grasp. Nonetheless, public trust in HBs can be enhanced by actively transforming certain empirical factors. Although it cannot guarantee that trust will necessarily arise, it can play a promotional role in the correct direction. Fostering public trust in HBs can be achieved through efforts to nurture the public’s propensity to trust, mold the trustworthiness of HBs themselves, establish structural safeguards for the trust environment, and strengthen public participation to engender a favorable “leap of faith.” Therefore, trust is not solely a matter for the public. It necessitates the awareness and reflection of the operators of HBs and policymakers and the concerted efforts of all societal parties.

Due to the inherent uncertainty of trust, a humble and open attitude should be maintained toward whether measures taken will yield effective trust. Furthermore, it is crucial to accurately comprehend the role of “distrust,” which may possess positive value for “trust,” depending on how it is addressed. Ultimately, it is vital to recognize that enhancing the acceptability of the public and society is not the ultimate objective. What is paramount is to establish a trustworthy foundation: ethical and normative development of HBs; activities and actions that benefit donors and society at large; reliable and adequate structural safeguards.

## Data Availability

The original contributions presented in the study are included in the article/Supplementary Material, further inquiries can be directed to the corresponding author.
